# A “Schizophotonic” All-In-One Nanoparticle Coating for Multiplexed SE(R)RS Biomedical Imaging**

**DOI:** 10.1002/anie.201403835

**Published:** 2014-08-27

**Authors:** Pasquale Iacono, Hazem Karabeber, Moritz F Kircher

**Affiliations:** Department of Radiology and Center for Molecular Imaging and Nanotechnology (CMINT), Memorial Sloan Kettering Cancer Center and Weill Cornell Medical CollegeNew York, NY 10065 (USA)

**Keywords:** fluorescence, gold, molecular imaging, nanoparticles, SERS

## Abstract

SERS nanoprobes for in vivo biomedical applications require high quantum yield, long circulation times, and maximum colloidal stability. Traditional synthetic routes require high metal–dye affinities and are challenged by unfavorable electrostatic interactions and limited scalability. We report the synthesis of a new near-IR active poly(*N*-(2-hydroxypropyl) methacrylamide) (pHPMA). The integration of various SERS reporters into a biocompatible polymeric surface coating allows for controlled dye incorporation, high colloidal stability, and optimized in vivo circulation times. This technique allows the synthesis of very small (<20 nm) SERS probes, which is crucial for the design of excretable and thus highly translatable imaging agents. Depending on their size, the “schizophotonic” nanoparticles can emit both SERS and fluorescence. We demonstrate the capability of this all-in-one gold surface coating and SERS reporter for multiplexed lymph-node imaging.

New molecular-based imaging techniques are consistently sought after for the real-time visualization of micro- and macroscopic biological processes.[1] New imaging probes and instrumentation can elucidate disease mechanisms in vivo, enable more precise imaging of the extent of diseases and aid in earlier detection. In addition, the use of non-invasive stimuli to excite and detect imaging probes is highly attractive. In the case of optical imaging probes, the use of near-infrared (NIR) light for this purpose has garnered much attention as a means of in vivo imaging because of its enhanced tissue penetration.[2] Nanoparticle technology has also contributed a multimodal aspect such that drug delivery and/or NIR-based photodynamic therapy can be adjoined onto a single nanoprobe, allowing for a holistic approach to disease management.[3]

Although NIR light-mediated imaging has been explored extensively with fluorescence-based techniques,[2] interest in Raman spectroscopy-based imaging,[4] specifically surface-enhanced Raman scattering (SERS),[5] is emerging as a result of the unprecedented signal intensity and signal specificity (detection of a unique “Raman fingerprint”) of the technique. SERS nanoprobes have been demonstrated to allow multimodal imaging of cancer, including SERS, photoacoustic, and magnetic resonance imaging (MRI); they can also achieve detection of cancerous tissues in vivo with microscopic precision.[6] Currently, single-particle SERS substrates are generally synthesized by adsorbing a dye directly onto a metal surface and then applying a surface coating to encapsulate the dye molecules and provide biocompatibility. To date, silication of a dye-adsorbed nanoparticle has been most effective in retaining in vivo SERS tag stability and biocompatibility.[5b] However, current silication methods depend on high dye–metal affinities, which can be limited by unfavorable electrostatic interactions. Sulfur-containing dyes with affinities to gold are available, but structure variation is limited and the dyes can be expensive. Scaling up the silication process can also often lead to size variation, aggregation, and uncontrollable dye incorporation and surface-charge manipulation.[7] Importantly, the silica layer also adds approximately double the size of the metal core, which limits the synthesis of SERS nanoparticles of the smaller size necessary for a variety of biomedical applications, for example, if renal or biliary clearance is intended.[8]

As an alternative to silicate layers, hydrophilic polymers as SERS substrate surface coatings are appealing because they can minimize nanoparticle size while retaining excellent biocompatibility and long intravascular circulation times desirable for efficient disease targeting.[5b] The polymers can also be tailored to be functionalized after they have been grafted onto the metal surface, allowing for subsequent particle modification. The facile syntheses of these SERS nanoprobes generally include treating dye-adhered metal nanoparticles with thiolated poly(ethylene glycol) or analogous polymers. However, competition for vacant binding sites can limit signal intensities and/or polymer grafting densities.[5b, 9] Therefore, a hydrophilic NIR-active polymer with the potential for further modification would be an ideal substrate for SERS applications, allowing for an all-encompassing surface coating for gold nanoparticles, retaining optimal (multi-)chromophore loading efficiency, minimizing size, and retaining its biocompatibility, water solubility, and circulative properties. Herein we report a class of hydrophilic NIR dye-loaded poly(*N*-(2-hydroxypropyl) methacrylamides) (pHPMA; Figure 1) and their application for gold nanoparticle-based SERS imaging of lymph nodes.

**Figure 1:**
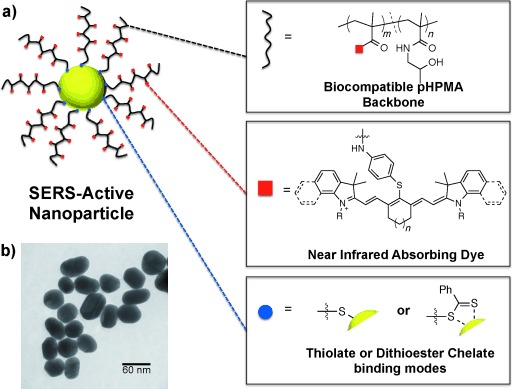
a) Composition of a SERS-active gold nanoparticle featuring an all-inclusive near-IR-active surface coating SERS substrate. b) Transmission electron microscopy (TEM) image of Au@IR-pHPMA.

Our synthetic strategy for preparing this unique NIR-active hydrophilic polymer stems from the modularity of its polymeric precursor, poly(pentafluorophenyl methacrylate) (pPFMA), which can be synthesized by reversible addition-fragmentation chain-transfer (RAFT) polymerization to produce well-defined, telechelic, and unimodal polymeric activated esters by bio-friendly metal-free methods.[10] Although the convenient dithioester terminus can be used as the chelating moiety that binds to the gold surface,[11] it can be reduced to a thiol with sodium borohydride to form thiol-terminated pPFMA (see Supporting Information) so a stronger covalent Au=S bond can be formed.[12] The labile ester bond of the air- and moisture-stable pPFMA can be easily cleaved in the presence of an amine and a base at ambient temperatures to give a multitude of microstructures (Scheme 1).[13] The pPFMA is also an alternative precursor to pHPMA, an established hydrophilic polymer with exceptional circulation properties and drug delivery capabilities.[14] We embarked on employing aniline-modified heptamethine cyanine dyes as our NIR-active conjugates, which were facilely synthesized by the treatment of commercially available IR-780, IR-806, or IR-820 with 4-aminothiophenol.[15] The dyes were then installed onto the polymer by aminolysis of the activated ester linkage on pPFMA in the presence of triethylamine. These reactions were monitored by taking aliquots and observing pentafluorophenol liberation by ^19^F NMR spectroscopy. Subsequently, addition of excess 2-hydroxypropylamine ensured complete conversion into the dye-modified pHPMAs (IR-pHPMA). Previously, methods used to create analogous polymers have included conjugating dyes to pHPMA polymers by hydrazide linkages, but these routes require extra synthetic steps, such as protection/deprotection chemistry, call for the use of expensive dyes, and do not include terminal sulfurous linkages suitable for gold bonding.[16]

**Scheme 1:**
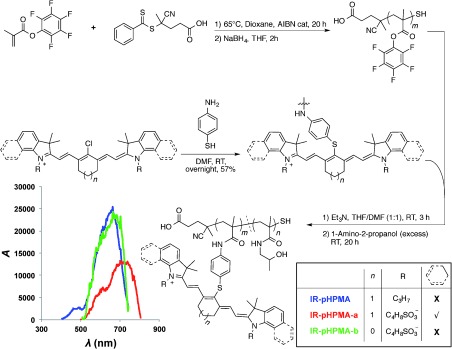
Synthesis and absorbance properties of NIR-active pHPMAs.

Scheme 1 includes the change in absorption spectra as the dyes are conjugated to the polymethacrylamide backbone. Considerable blue-shifts in the dyes’ excitation wavelengths upon conjugation are observed. This phenomenon has been reported for NIR dye-modified poly(isobutylene-*alt*-maleic anhydride)s,[17] polyamides,[18] and polyethyleneimines.[19] Because the absorption change is hypsochromic, head-to-tail interactions (H-aggregates) amongst appended dye molecules are suggested to occur.[18] Additionally, compared to the free dye, we observed enhanced emissions after the dyes were appended to the polymer backbone. In addition to polymer-induced constraints, we hypothesize that the phenyl-based dye–polymer bridge may also provide an aromatic stacking route to provoke an apparent aggregation-induced enhancement,[20] which we aim to investigate in future studies.

In the presence of citrate-stabilized gold nanoparticles, the thiol-terminated polymers can bind to the nanoparticle through thermodynamically stable sulfur–gold bonds, yielding the SERS-active probes (Au@IR-pHPMA) with diameters (55±11) nm as determined by TEM analysis. Additionally, atomic force microscopy (AFM) of the nanoparticles demonstrates a uniform surface coating, based on tapping-mode phase imaging techniques (Figure S13 in the Supporting Information).[21] Adhesion-mapping methods[22] were also performed to differentiate probe–gold nanoparticle versus probe–Au@IR-pHPMA interactions by surface-scanned force measurements. The resulting mean-adhesion values were observed to be consistent over multiple nanoparticles with both gold-based and high-resolution silicon nitride-based probes (Figure S14).

Figure 2 shows the differences in SERS spectra of the three different nanoprobes as well as their corresponding SERS images, attesting to their in vitro multiplexing capabilities. Because IR-pHPMA-a absorbs closer to the wavelength of the 785 nm laser (ca. 720 nm) than the other two analogues by approximately 60 nm, we observed a much stronger Raman signal as a result of resonance Raman scattering (SERRS). Figure 3 a shows how a slight variance in dye content influences the Raman spectra using a 785 nm laser at 5 mW laser power. As smaller gold nanoparticles are surface coated with IR-pHPMA, the intrinsic fluorescence becomes more prominent (Figure 3 b,c) while shrouding SERS signatures owing to distal polymer-appended dye molecules that are not affected by the gold’s quenching electric field.[23] Because dye molecules are randomly conjugated throughout the polymer, electromagnetic SERS enhancement is optimal within 2–3 nm of the gold surface and decreases as an inverse function of the radius of the gold sphere and dye–surface distance.[24] Some lower-frequency background fluorescence can be imaged with Au@IR-pHPMA even with the larger gold nanospheres, but the bulk of the polymers’ inherent fluorescence, which overlaps with the Raman spectral window, is expectedly quenched by the gold nanoparticle. Figure 3 e shows that the free polymer exhibits a notable near-infrared fluorescence (NIRF) and no SERS signal, whereas its gold-bound analogue exhibits a weakly visible NIRF signal and a highly amplified SERS signal. We have coined the term “schizophotonic” to describe the polymers’ ability to split emission types while anchored to the gold surface. Furthermore, to our knowledge, the ability to produce SERS-active nanoparticles as small as 19 nm has not been previously reported.

**Figure 2:**
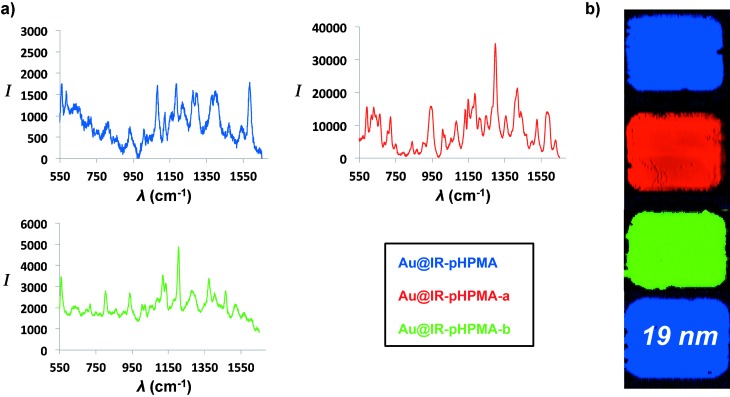
a) Raman spectra (1 nm aqueous colloid, 1 mW laser power [785 nm], 1 s acquisition time), and b) corresponding multiplexed SERS images of Au@IR-pHPMA and its analogues, including its 19 nm gold-core variant.

**Figure 3:**
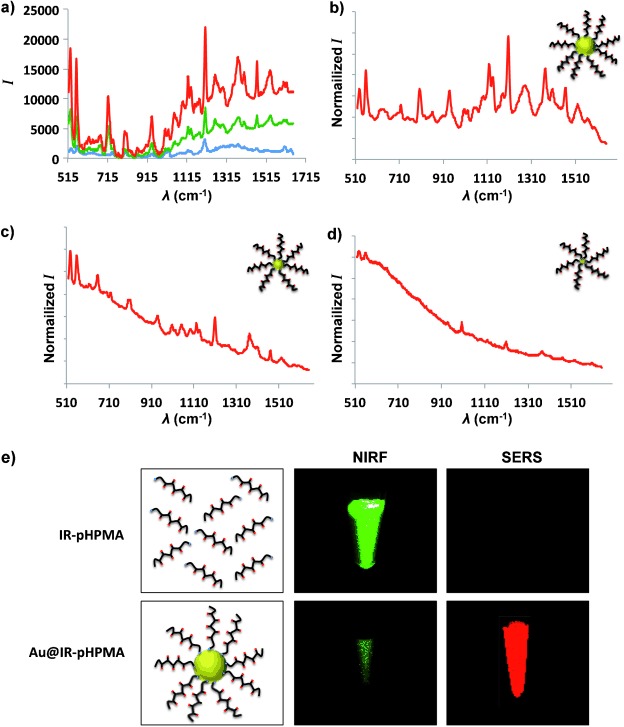
Raman properties of Au@IR-pHPMA. a) Baseline-corrected (for signal comparison) SERS spectra (5 mW laser power [785 nm], 1 s acquisition time) of Au@IR-pHPMA (1 nm aqueous colloid) with polymer-dye loadings of 1.8 (blue), 2.4 (green), and 3.2 mol % (red). Normalized SERS spectra (1 mW, 1 s acquisition time) of Au@IR-pHPMA (1 nm aqueous colloid, 3.2 mol % polymer–dye loading) with b) 55 nm, c) 33 nm, and d) 19 nm gold cores. e) SERS and near-infrared fluorescence (NIRF) images of Eppendorf vials containing aqueous solutions of IR-pHPMA (4 mg mL^−1^) and Au@IR-pHPMA (3 nm) nanoparticles.

With these viable SERS nanoprobes in hand, we sought to explore their capacity for imaging to identify the first (sentinel) lymph nodes after subcutaneous probe injection, a process that is analogous to techniques used routinely during the treatment of most types of cancer.[25] Techniques such as radioactive tracer imaging and NIR fluorescence imaging, are current methods for sentinel lymph node mapping.[25] SERS imaging could prove vital to a surgeon in mapping these lymph nodes with improved accuracy, because it provides higher resolution than radioactive methods, and higher signal specificity (no autofluorescence) than fluorescence methods. In our experiments, non-tumor bearing nude mice were administered Au@IR-pHPMA in an MES buffered solution (10 μL, 3 nm, pH 7.3) in the paw of the right forelimb, and sentinel lymph node uptake was monitored at 2 and 24 h. At 2 h, SERS imaging demonstrated that the nanoparticles were draining towards the sentinel lymph node, with signal being observed both in the injection site of the forelimb and in the lymph node itself (Figure 4 a). At 24 h we observed strong SERS signal in the axillary lymph node and slight signal in the forelimb, indicating that the particles had drained almost completely from the lymphatic ducts into the lymph node. As a proof of concept, we also synthesized a variant of Au@IR-pHPMA where the terminal anchoring group for the polymer on the gold surface was the untreated dithioester (DTE) chelate. Upon administration of these analogues (Au@IR-pHPMA-DTE), we noticed that although SERS signal was observed in the axillary lymph node at 2 h, negligible signal was observed at 24 h (Figure S16). We hypothesized that this signal diminishment was caused by particle degradation by disassociation of polymer dithioester group from the gold surface, suggesting the necessity for a covalent Au–S linkage to uphold longer term in vivo stability.

**Figure 4:**
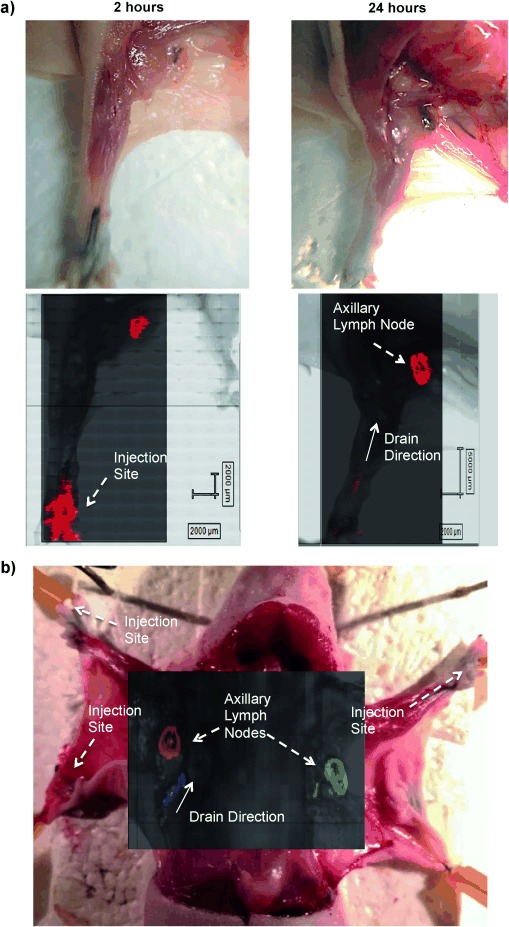
a) Photographs (top) and SERS image (bottom) of axillary lymph nodes and forelimb after injections of Au@IR-pHPMA (3 nm, 10 μL). b) Photograph and overlying SERS image of axillary lymph nodes and surrounding tissue 24 h after multisite injections of Au@IR-pHPMA (red), Au@IR-pHPMA-a (green), and Au@IR-pHPMA-b (blue) (3 nm, 10 μL each).

Finally, we sought to investigate the in vivo multiplexing capability of the three nanoparticles by separately injecting each of them into three different sites and observing their distinct lymphatic drainage pathways (Figure 4 b). As Au@IR-pHPMA and Au@IR-pHPMA-a were injected in opposite paws, they accumulated separately in opposite axillary lymph nodes (Figure 4 b red, green). Au@IR-pHPMA-b was injected into the right mammary fat pad, and was still draining within the lymphatic ducts (Figure 4 b blue) at the time of imaging because of the slower drainage speed in these alternate ducts.

In summary, we have demonstrated that a dye-modified hydrophilic polymer can be utilized as a viable all-inclusive surface coating and multiplex-capable SERS substrate for gold nanoparticles. Gold nanoparticles coated with polymer which is bound by covalent gold–thiolate bonds show longer in vivo signal stability in a 24 hour period compared to coated particles assembled with noncovalent gold–dithioester chelate interactions, and allow precise detection of lymph nodes, an important application in oncology. The particles’ stability can be attributed to the robust amide conjugates of the dyes on the polymer, the stability of the biocompatible polymethacrylamide polymer itself, and, ultimately, the polymers’ stable covalent bond with the gold nanoparticle surface. The ability to produce SERS-active nanoparticles smaller than 20 nm can also open up important avenues in biomedical applications where biliary or renal excretion is required for clinical translation.[8] In addition, the so-called “schizophotonic” behavior of the polymers provide a means of achieving simultaneous SERS and (semi-quenched) fluorescence in the smaller particles, which can be advantageous in applications where dual SERS-NIRF signal is desired.[26]
