# Supplementary material for: A “Schizophotonic” All-In-One Nanoparticle Coating for Multiplexed SE(R)RS Biomedical Imaging
Source: Angew Chem Int Ed Engl. 2014 Aug 27;53(44):11756–61. doi: 10.1002/anie.201403835 (PMC4389888; doi:10.1002/anie.201403835)
Supplement: Supplementary file 1 [file anie0053-11756-sd1.pdf]

Angewandte Chemie
Eine Zeitschrift der Gesellschaft Deutscher Chemiker

Supporting Information
© Wiley-VCH 2014
69451 Weinheim, Germany

A "Schizophotonic" All-In-One Nanoparticle Coating for Multiplexed SE(R)RS Biomedical Imaging**

*Pasquale Iacono, Hazem Karabeber, and Moritz F. Kircher**

anie_201403835_sm_miscellaneous_information.pdf

Table of Contents

A. Materials and Methods 2
Synthesis of IR-780-NH2 3
Synthesis of IR-820-NH_2 4
Synthesis of IR-806-NH_2 4
Synthesis of Poly(pentafluorophenyl methacrylate) (pPFMA) 5
Synthesis of Thiol-Terminated Poly(pentafluorophenyl methacrylate) 5
Synthesis of IR-780-NH2-conjugated Poly(*N*-(2-hydroxypropyl) methacrylamide) (IR-pHPMA) 6
Synthesis of Au@IR-pHPMA 7
AFM imaging of Au@IR-pHPMA 7
Cell Viability Assay 8
In vivo Raman imaging of Au@IR-pHPMA 9
B. Supplementary Data 10
Figure S1. ^{1}H NMR Spectrum (500 MHz, $CDCl_3$) of IR-780-NH_2 10
Figure S2. ^{13}C NMR Spectrum(126 MHz, $CDCl_3$) of IR-780-NH_2 11
Figure S3. ^{1}H NMR Spectrum (600 MHz, CD_3OD) of IR-820-NH_2 12
Figure S4. ^{13}C NMR Spectrum (126 MHz, CD_3OD) of IR-820-NH_2 13
Figure S5. ^{1}H NMR Spectrum (600 MHz, CD_3OD) of IR-806-NH_2 14
Figure S6. ^{13}C NMR Spectrum(126 MHz, CD_3OD) of IR-806-NH_2 15
Figure S7. ^{1}H NMR Spectrum (500 MHz, CD_3OD) of IR-pHPMA 16
Figure S8. ^{13}C NMR Spectrum (150 MHz, CD_3OD) of IR-pHPMA 17
Figure S9. ^{1}H NMR Spectrum (500 MHz, CD_3OD) of IR-pHPMA-a 18
Figure S10. ^{13}C NMR Spectrum (150 MHz, CD_3OD) of IR-pHPMA-a 19
Figure S11. ^{1}H NMR Spectrum (500 MHz, CD_3OD) of IR-pHPMA-b 20
Figure S12. ^{13}C NMR Spectrum(150 MHz, CD_3OD) of IR-pHPMA-b 21
Figure S13. Tapping mode AFM phase images of a) bare gold nanoparticles and b) Au@IR-pHPMA on AP-Mica substrate with an SSS-SEIHR 2 nm high-resolution probe. 22
Figure S14. Representative histograms of AFM adhesion measurements and accompanying adhesion maps of a) bare gold nanoparticles and b) Au@IR-pHPMA using a gold AFM probe. Histogram of adhesion force measurements and accompanying adhesion maps for c) bare gold nanoparticles and d) Au@IR-pHPMA using a silicon nitride probe. 23
Figure S15. Curve portraying HEK-293 cell viability after 24 h incubation with varying concentrations of Au@IR-pHPMA. 24
Figure S16. Photographs (top) and SERS images (bottom) of axillary lymph nodes and fore limbs after injection of Au@IR-pHPMA-DTE 25
C. Supplementary References 26

A. Materials and Methods

All reagents were purchased from Sigma-Aldrich and were used without further purification. Citrate-stabilized gold nanospheres were prepared by previously reported methods.[1] Nuclear magnetic resonance (NMR) spectra were recorded on either a Bruker Avance III 500 MHz instrument or a Bruker Avance III 600 MHz instrument equipped with a cryo-probe at 24 °C and were referenced versus residual non-deuterated solvent shifts (^{1}H, ^{13}C). Mass spectra were obtained at the Memorial Sloan Kettering Cancer Center Analytical Core Facility. Polymer polydispersities and molecular weights were determined by gel permeation chromatography in tetrahydrofuran (with 0.01 wt. % di-*tert*-butylhydroxytoluene (BHT) radical inhibitor). Polymer samples were eluted through two ViscoGel columns in series, I-MBMMW-3078 and I-MBLMW-3078, using a Viscotek GPCMax VE2001 coupled with a Viscotek TDA305 Triple Detector Array, normalized to a 99,000 Da polystyrene standard. Particle concentrations were determined by a NS500 nanoparticle tracking analysis (NTA) instrument (Nanosight, Duxbury, MA). Transmission electron microscopy images were acquired on carbon grids (Ted Pella, Inc.) on a Jeol 1200 EX microscope. Absorption spectra were obtained on a Tecan Infinite M1000 PRO fluorometer. Fluorescence imaging was performed on a LI-COR Odyssey through an 800 nm bandpass filter. Atomic force microscopy was performed on an Asylum Research MFP-3D-BIO microscope with an Olympus TR400PB gold probe and a Bruker DNP-S Silicon Nitride probe for high-resolution force mapping and a NanoSensors SSS-SEIHR 2 nm probe for high-resolution tapping mode imaging. The nanoparticles were adhered to positively charged AP-Mica (as prepared by common techniques) for 10-30 minutes before washing with ddH_2O and drying under nitrogen.

For adhesion mapping measurements,[2] cantilever stiffness was calibrated using the Thermal Tune method provided by the Asylum Research system software. 16 x 16 points were acquired with the gold probes and 256 x 256 points were acquired with the high-resolution DNP-S tips over a 15 x 15 nm and 1 µm x 1 µm surface area, respectively. The measurements were fit to a Gaussian curve and mean adhesions were calculated from the curve. Raman spectra and images were obtained with an *InVia* Raman microscope with StreamLine™ Plus upgrade (Renishaw Inc., Hoffman Estates, IL) as previously described.[3] The synthesis of **Au@IR-pHPMA-DTE** followed the same protocols as **Au@IR-pHPMA** in the absence of $NaBH_4$ treatment for retention of the terminal dithioester (DTE) moiety.

Synthesis of IR-780-NH2

Following a previously reported procedure,[4] IR-780 (498 mg, 0.75 mmol) was treated with 4-aminothiophenol (187 mg, 1.49 mmol) in DMF (10 mL) and stirred overnight. The solution was concentrated and the crude mixture was separated via silica gel column chromatography (CH_2Cl_2/MeOH 3:1). Fractions containing the desired compound were recrystallized from MeOH/Et_2O to afford dark green/gold crystals (325 mg, 57% yield) ^{1}H NMR (500 MHz, $CDCl_3$) δ 8.76 (d, *J* = 14.2 Hz, 2H), 7.39-7.28 (m, 4H), 7.20 (t, *J* = 7.4 Hz, 2H), 7.11 (d, *J* = 7.9 Hz, 2H), 7.01 (d, *J* = 8.6 Hz, 2H), 6.64 (d, *J* = 8.6 Hz, 2H), 6.18 (d, *J* = 14.2 Hz, 2H), 4.09 (t, *J* = 7.3 Hz, 4H), 2.72 (t, *J* = 6.2 Hz, 4H), 2.00 (p, *J* = 6.2 Hz, 2H), 1.89 (m, *J* = 7.4 Hz, 4H), 1.55 (s, 12H), 1.05 (t, *J* = 7.4 Hz, 6H). ^{13}C NMR (126 MHz, $CDCl_3$) δ 172.28, 154.43, 146.52, 145.33, 142.37, 141.13, 134.09, 129.98, 128.63, 128.18, 125.02, 124.65, 122.20, 116.31, 110.69, 101.38, 49.21, 46.22, 28.06,

26.70, 20.87, 11.70. HRMS (ESI): m/z (M)+ calcd for $C_{42}H_{50}N_3S$, 628.3725; found, 628.3719.

Synthesis of IR-820-NH$_2$

Similarly to the synthesis of **IR-780-NH$_2$**, IR-820 (1.00 g, 1.17 mmol) was treated with 4-aminothiophenol (295 mg, 2.35 mmol) in DMF (15 mL) and stirred overnight. The solution was concentrated and precipitated out of diethyl ether three times to afford a red powder (428 mg, 39% yield) ^{1}H NMR (600 MHz, CD_3OD) δ 8.93 (d, *J* = 14.1 Hz, 1H), 8.20 (d, *J* = 8.5 Hz, 1H), 8.06 - 7.95 (m, 2H), 7.64 (t, *J* = 8.4 Hz, 2H), 7.49 (t, *J* = 7.5 Hz, 1H), 7.29 (d, *J* = 8.4 Hz, 1H), 7.00 (d, *J* = 8.2 Hz, 1H), 6.39 (d, *J* = 14.1 Hz, 1H), 4.32 (t, *J* = 7.5 Hz, 2H), 2.95 - 2.80 (m, 4H), 2.13 - 1.89 (m, 6H), 1.85 (s, 6H). ^{13}C NMR (126 MHz, CD_3OD) δ 172.28, 154.43, 146.52, 145.33, 142.37, 141.13, 134.09, 129.98, 128.63, 128.18, 125.02, 124.65, 122.20, 116.31, 110.69, 101.38, 49.21, 46.22, 28.06, 26.70, 20.87, 11.70. HRMS (ESI): m/z (M)+ calcd for $C_{52}H_{58}N_3O_6S_3$, 916.3488; found, 916.3481.

Synthesis of IR-806-NH$_2$

Similarly to the synthesis of **IR-820-NH$_2$**, IR-806 (1.00 g, 1.36 mmol) was treated with 4-aminothiophenol (340 mg, 2.71 mmol) in DMF (15 mL) and stirred overnight. The solution was concentrated and precipitated out of diethyl ether three times to afford a blue solid (180 mg, 16% yield) ^{1}H NMR (600 MHz, CD_3OD) δ 8.76 (d, *J* = 14.2 Hz, 2H), 7.39-7.28 (m, 4H), 7.20 (t, *J* = 7.4 Hz, 2H), 7.11 (d, *J* = 7.9 Hz, 2H), 7.01 (d, *J* = 8.6 Hz, 2H), 6.64 (d, *J* = 8.6 Hz, 2H), 6.18 (d, *J* = 14.2 Hz, 2H), 4.09 (t, *J* = 7.3 Hz, 4H), 2.72 (t,

J = 6.2 Hz, 4H), 2.00 (p, J = 6.2 Hz, 2H), 1.89 (m, J = 7.4 Hz, 4H), 1.55 (s, 12H), 1.05 (t, J = 7.4 Hz, 6H). ^{13}C NMR (126 MHz, CD_3OD) δ 175.26, 152.75, 146.61, 141.13, 135.14, 135.03, 134.65, 133.45, 131.86, 131.13, 129.40, 129.04, 128.74, 126.13, 123.41, 123.14, 120.69, 112.26, 102.21, 52.36, 51.88, 45.18, 27.88, 27.63, 27.41, 23.58, 22.29. HRMS (ESI): m/z (M)+ calcd for $C_{43}H_{52}N_3O_6S_3$, 802.3018; found, 802.3010.

Synthesis of Poly(pentafluorophenyl methacrylate) (pPFMA)

Poly(pentafluorophenyl methacrylate) was synthesized according to a previously reported method.[5] Briefly, a solution of the chain transfer agent (CTA) 4-cyano-4-((thiobenzoyl)sulfanyl)pentanoic acid (200 μmol), AIBN (8:1 CTA:AIBN), and pentafluorophenyl methacrylate (15 g, 60 mmol) in dioxane (20 mL) was deoxygenated by three freeze-pump-thaw cycles. The solution was then heated to 65 °C and stirred under nitrogen for 20 h. The polymer was precipitated out of hexanes and filtered. The material was purified by repeated precipitations from a THF/hexanes solution (x 2), affording a light pink powder (M_n 81.3 kDa, PDI 1.01).

Synthesis of Thiol-Terminated Poly(pentafluorophenyl methacrylate)

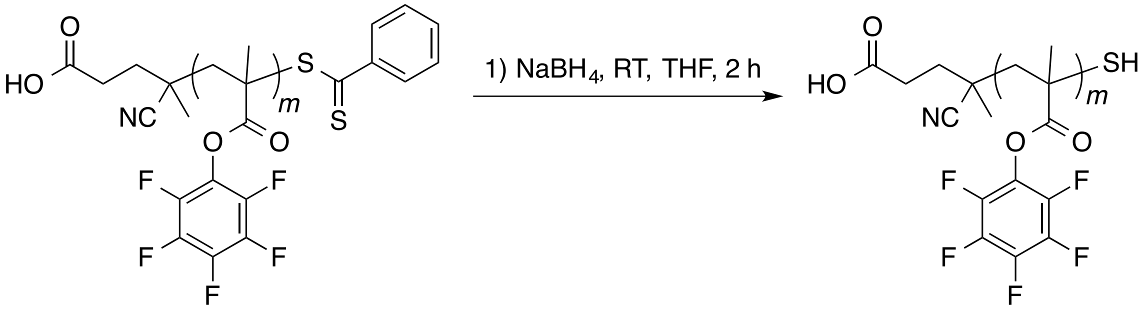

To achieve complete conversion of the terminal dithioester to the thiol, a solution of pPFMA (200 mg) in THF (15 mL) was treated with $NaBH_4$ (284 mg, 7.5 mmol) in 2 mL

of methanol and stirred at ambient temperature for 2 hr. The solution was reduced by rotary evaporation to 2 mL and precipitation of the polymer was induced by addition of excess methanol. The polymer was purified by redissolution in THF and methanolic-induced precipitation and dried in vacuo overnight to afford a white solid (>99% yield). Thiol production was verified and monitored using Ellman's reagent.

Synthesis of IR-780-NH2-conjugated Poly(*N*-(2-hydroxypropyl) methacrylamide) (IR-pHPMA)

The following procedure was also applied for the synthesis of **IR-pHPMA-DTE**, **IR-pHPMA-a**, and **IR-pHPMA-b**. Under nitrogen, poly(pentafluorophenyl methacrylate) (200 mg) was treated with 60 mg (0.2 molar eq to reactive ester groups) of **IR-780-NH_2** and an excess of triethylamine (220 μL) in a THF/DMF (1:1) solution (7 mL). Aminolysis was monitored using ^{19}F NMR spectroscopy by observing the liberation of pentafluorophenol. After 3 h, an excess of 1-amino-2-propanol (124 μL) was added to ensure full conversion of the remaining PFMA groups. The polymer was then precipitated into diethyl ether. Multiple precipitations out of DMF/Et_2O followed by drying *in vacuo* afforded the desired polymer as a dark green solid (70 mg). Polymers were characterized by ^{1}H/^{13}C NMR spectroscopy (Figures S7-S12). Dye content for **IR-pHPMA(-DTE)** (7.2 mol%), **IR-pHPMPA-a** (5.7 mol%), and **IR-pHPMA-b** (10.3 mol%) was determined by ^{1}H NMR spectroscopy.

Synthesis of Au@IR-pHPMA

The following procedure was also applied for the synthesis of **Au@IR-pHPMA-DTE**. An aqueous solution containing **IR-pHPMA** (10 mg/L) and citrate stabilized gold nanospheres (0.1 nM) was agitated for 3 days in the absence of light. The particles were then isolated by centrifugation, washed with ethanol (x 2) and with water. The final particle suspension in water was then stored in the dark at 4 °C for future studies.

AFM imaging of Au@IR-pHPMA

First, atomic force microscopy (AFM) "tapping mode" was employed to compare the phase images between polymer-coated and bare nanoparticles (Fig. S13). AFM phase images indicate tip energy dissipation, and have been used to identify heterogeneity in polymer surfaces;[2b] this method would indicate uneven coverage of the polymer for artifacts larger than 2 nm (the probe resolution). The phase images were extremely uniform on the surfaces of all of the polymer-coated nanoparticles, which, while not directly proving the presence of the polymer coating itself, does suggest that the surface was extremely homogeneous, both within a single nanoparticle, and amongst the entire population of nanoparticles (Fig. S13).

Next, the presence and distribution of the polymer coating was tested with an AFM force spectroscopy method to interrogate the adhesion force between the probe-tip and the nanoparticle (Fig. S14). If the bare gold and polymer-coated nanoparticles exhibited unique adhesion properties between tips of different materials, we could be certain that the polymer was present, and was affecting the behavior of the tip-sample interaction when compared to the control. First, a gold-coated probe was used to conduct 15x15 nm

force maps on the top face of individual nanoparticles to ensure an even contact with the probe tip. The mean adhesion of the gold-gold interaction was 1.65 ± 0.20 nN and the mean adhesion of the gold-**Au@IR-pHPMA** interaction was 2.68 ± 0.14 nN (n=7) (Fig. S14 a,b). The consistency in the differences was encouraging, so a force map of multiple nanoparticles in a single field would be ideal for comparison. This time, we used a silicon nitride probe across a 1x1 μm area that contained several dozen nanoparticles for each group. A total of 65,536 force measurements were taken at each point of a 256x256 grid. Comparison of the extremely robust histograms yielded differences again, but the silicon-nitride-probe adhesion interaction was inverted compared to the gold-probe adhesion interaction. This is due to gold's higher affinity for the methacrylamide polymer versus that of a more inert silicon nitride. The mean adhesion for **Au@IR-pHPMA** was 3.40 ± 0.52 nN and for bare gold was 4.18 ± 0.98 nN (Fig. S14 c,d).

Cell Viability Assay

HEK-293 cells, derived from human embryonic kidneys, were cultured in Dulbecco's Modified Eagle's Medium with 10% fetal bovine serum and incubated for 24 hours with **Au@IR-pHPMA** with a particle concentration ranging from 0 to 250 pM. WST-1 reagent (Roche, Mannheim, Germany) was then added into the medium (1:10). After one hour, absorbance intensities at 440 nm were measured for each sample, as measured by a Safire Multi-detection Microplate Reader (Tecan, Durham, NC, USA).

***In vivo* Raman imaging of Au@IR-pHPMA**

Animal studies were conducted in compliance with protocols approved by the Institutional Animal Care and Use Committee of Memorial Sloan-Kettering Cancer Center. A 10 microliter suspension of **Au@IR-pHPMA** in 2-(N-morpholino)ethanesulfonic acid (MES) buffer (pH 7.3) with a particle concentration of 3 nM was injected in the paw of the forelimb of non-tumor bearing nude mice (athymic Ncr-nu/nu, NCI). After 24 h the mice were sacrificed by carbon dioxide asphyxiation and positioned under the microscope for Raman imaging. A semiconductor diode near-infrared laser operating at 785 nm was used as the excitation source, with a laser power of ~100 mW being measured at the tissue surface. We obtained Raman images using the Renishaw StreamLine™ Plus scanning software. We used a computer-controlled x-y translation stage to scan over the area of interest, using an integration time of 1.5 s, a 5x magnification lens, and a slit size of 400 µm. Raman spectra were analyzed with Wire 2.0 Software (Renishaw).

B. Supplementary Data

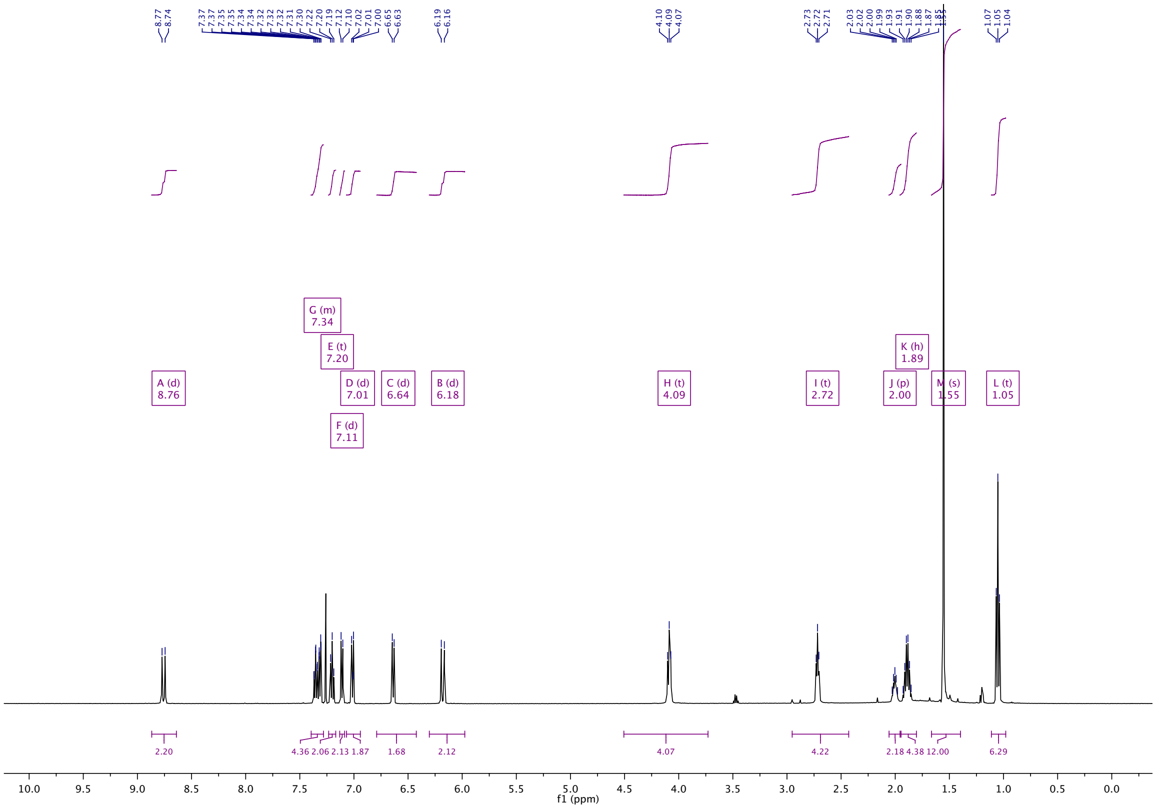

Figure S1. ^{1}H NMR Spectrum (500 MHz, $CDCl_3$) of IR-780-NH_2

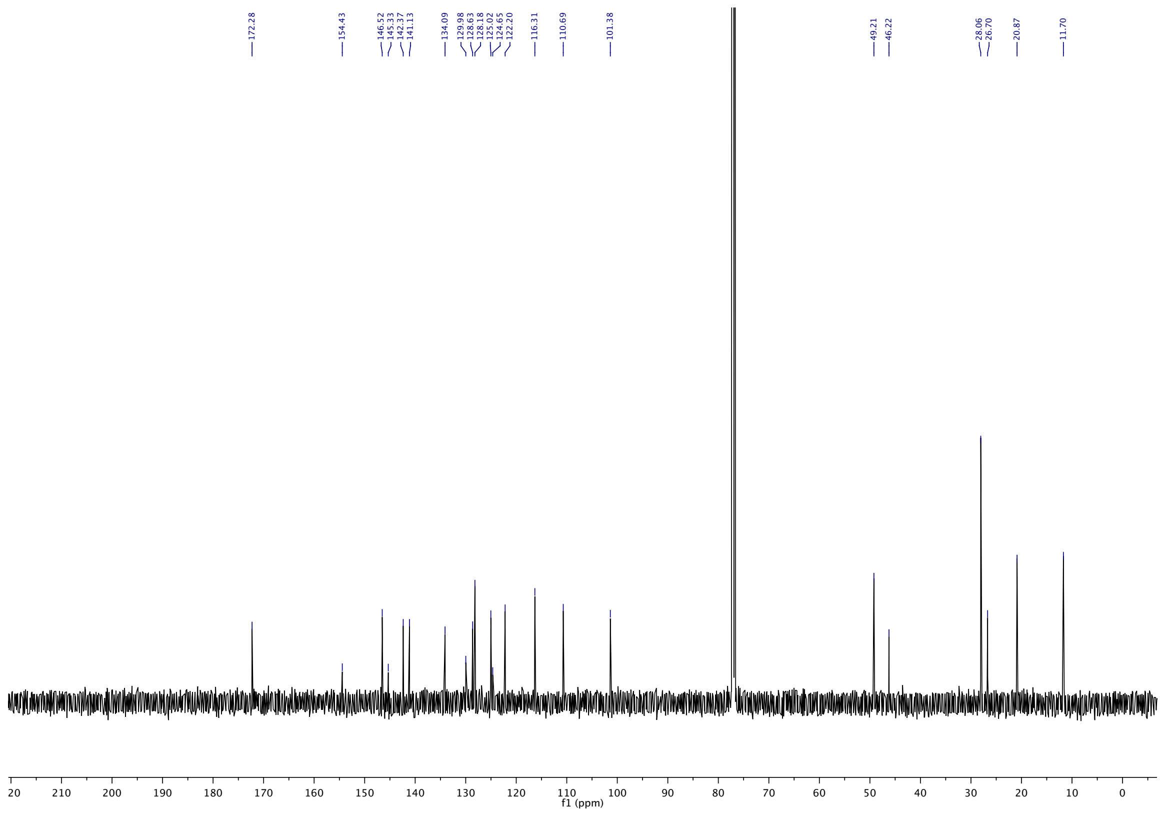

Figure S2. ^{13}**C NMR Spectrum(126 MHz, $CDCl_3$) of IR-780-NH_2**

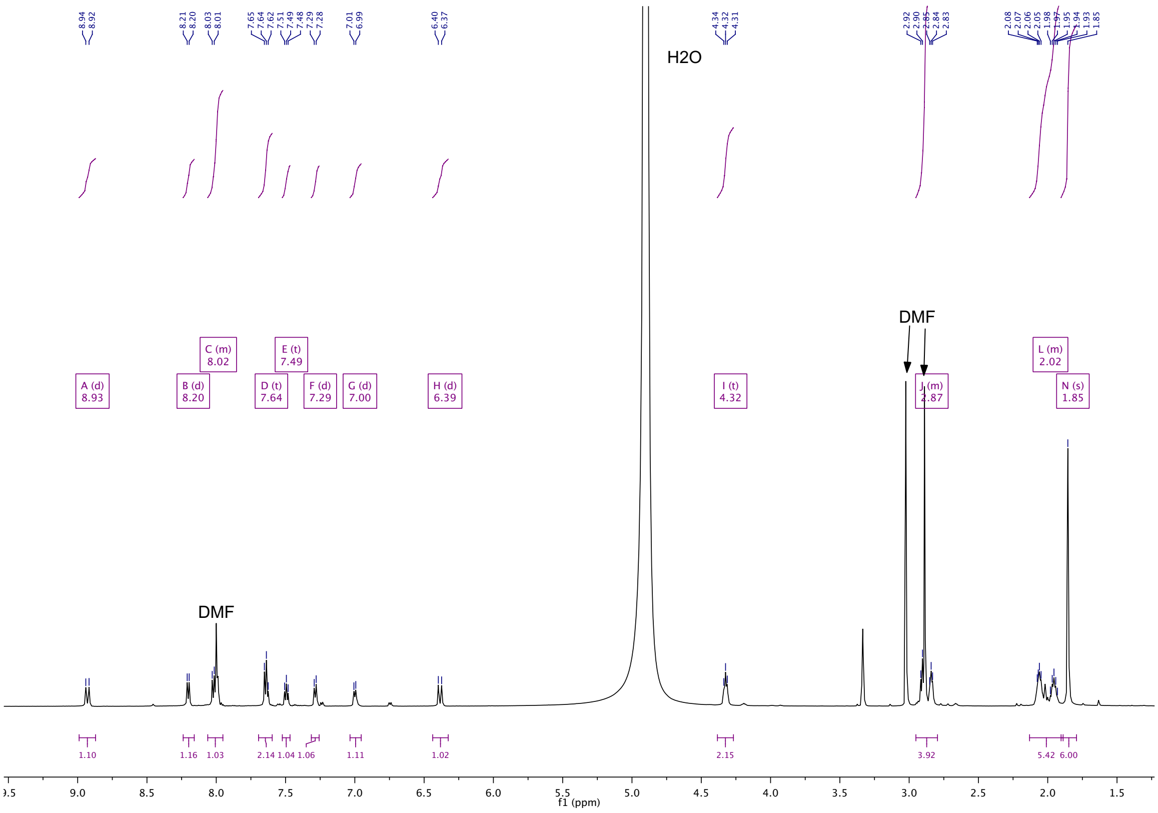

Figure S3. ^{1}H NMR Spectrum (600 MHz, CD_3OD) of IR-820-NH_2

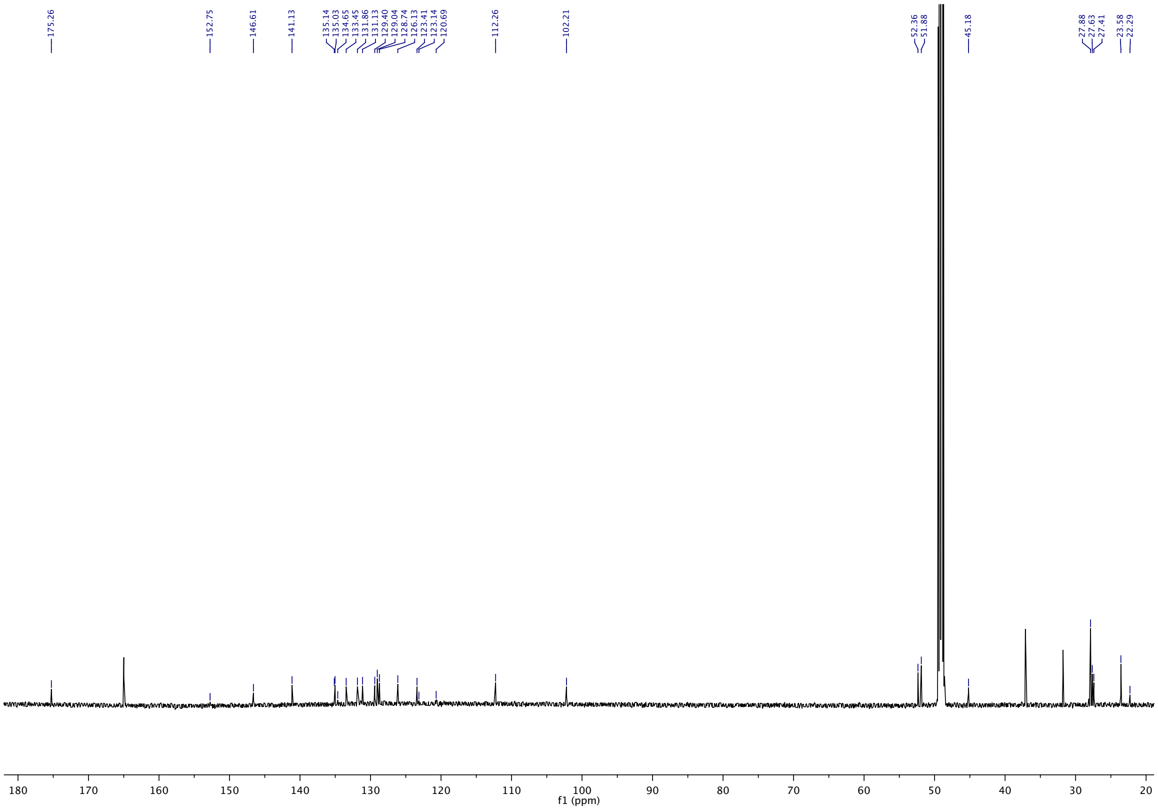

Figure S4. ^{13}C NMR Spectrum (126 MHz, CD_3OD) of IR-820-NH_2

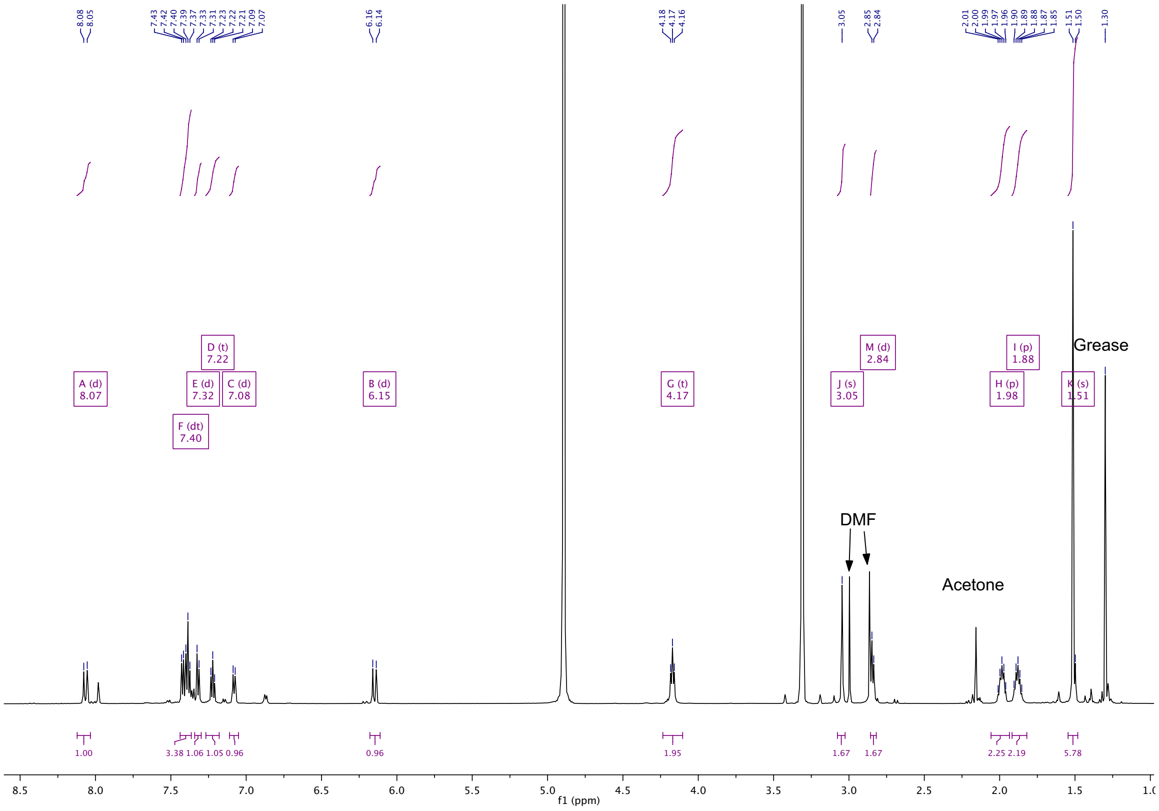

Figure S5. ^{1}H NMR Spectrum (600 MHz, CD_3OD) of IR-806-NH_2

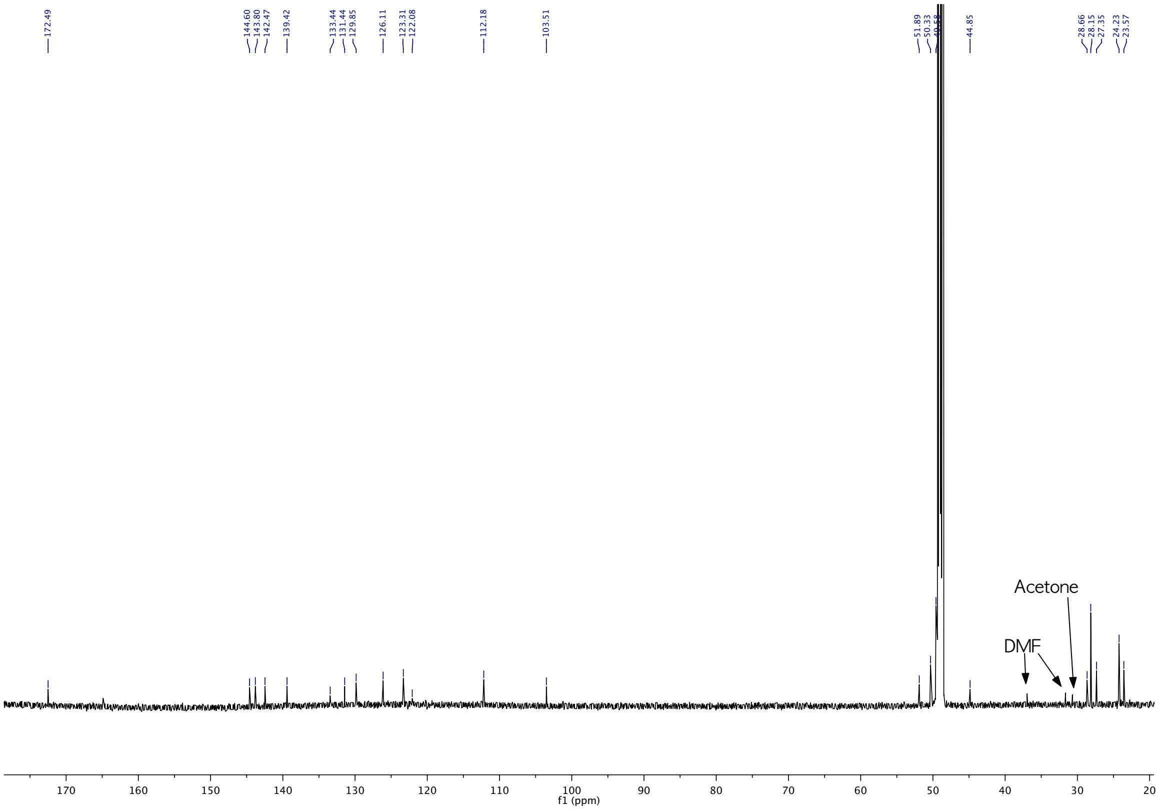

Figure S6. ^{13}C NMR Spectrum(126 MHz, CD_3OD) of IR-806-NH_2

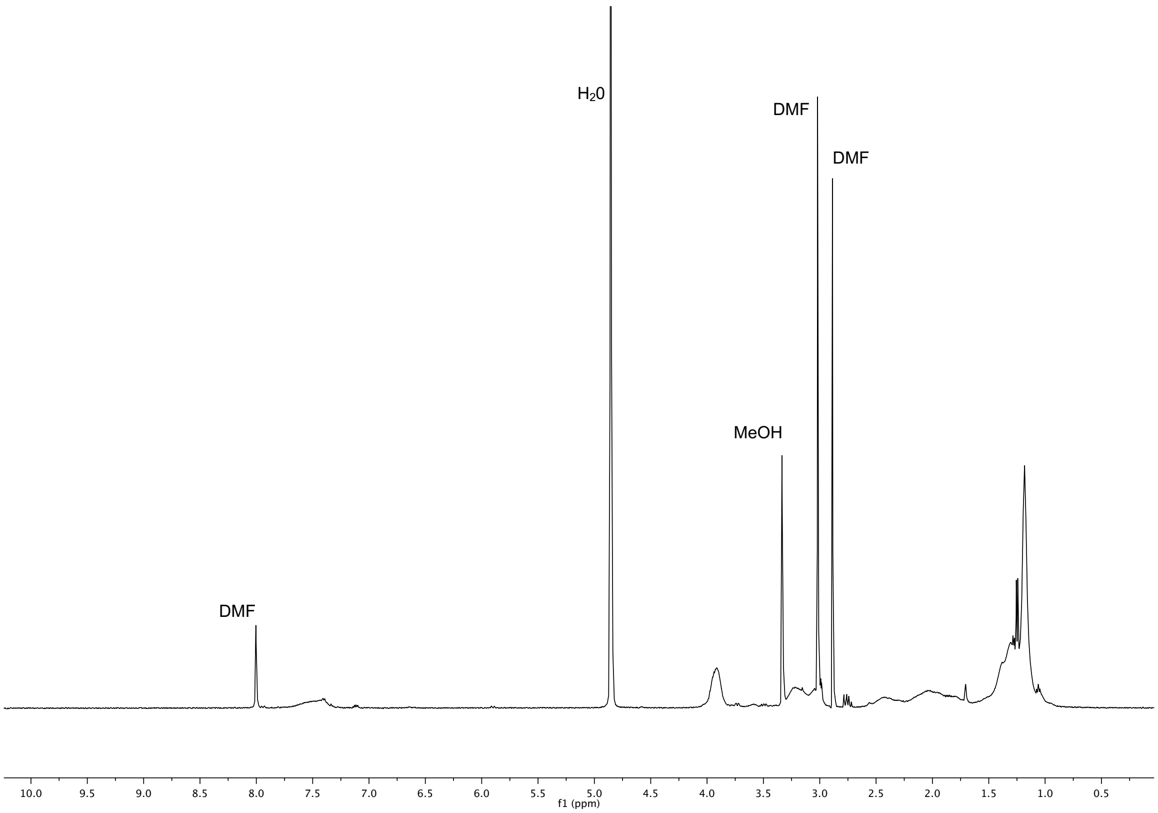

Figure S7. ^{1}H NMR Spectrum (500 MHz, CD_3OD) of IR-pHPMA

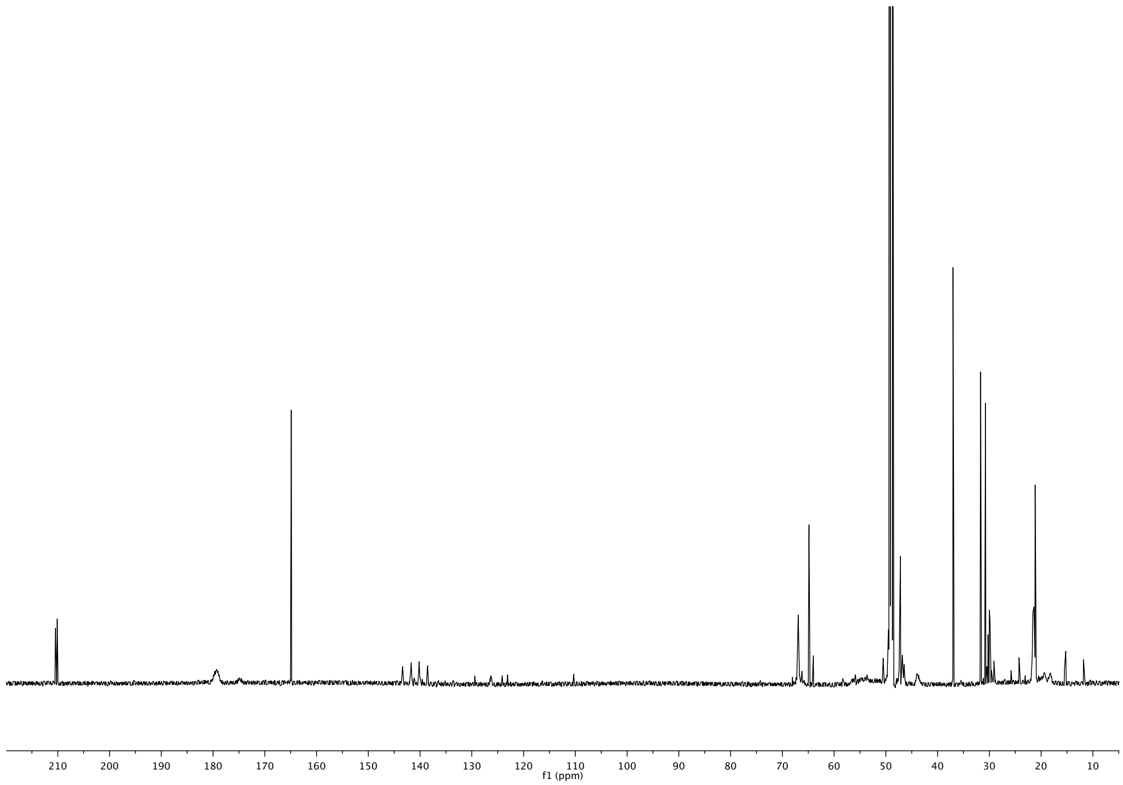

Figure S8. ^{13}**C NMR Spectrum (150 MHz,** CD_3OD**) of IR-pHPMA**

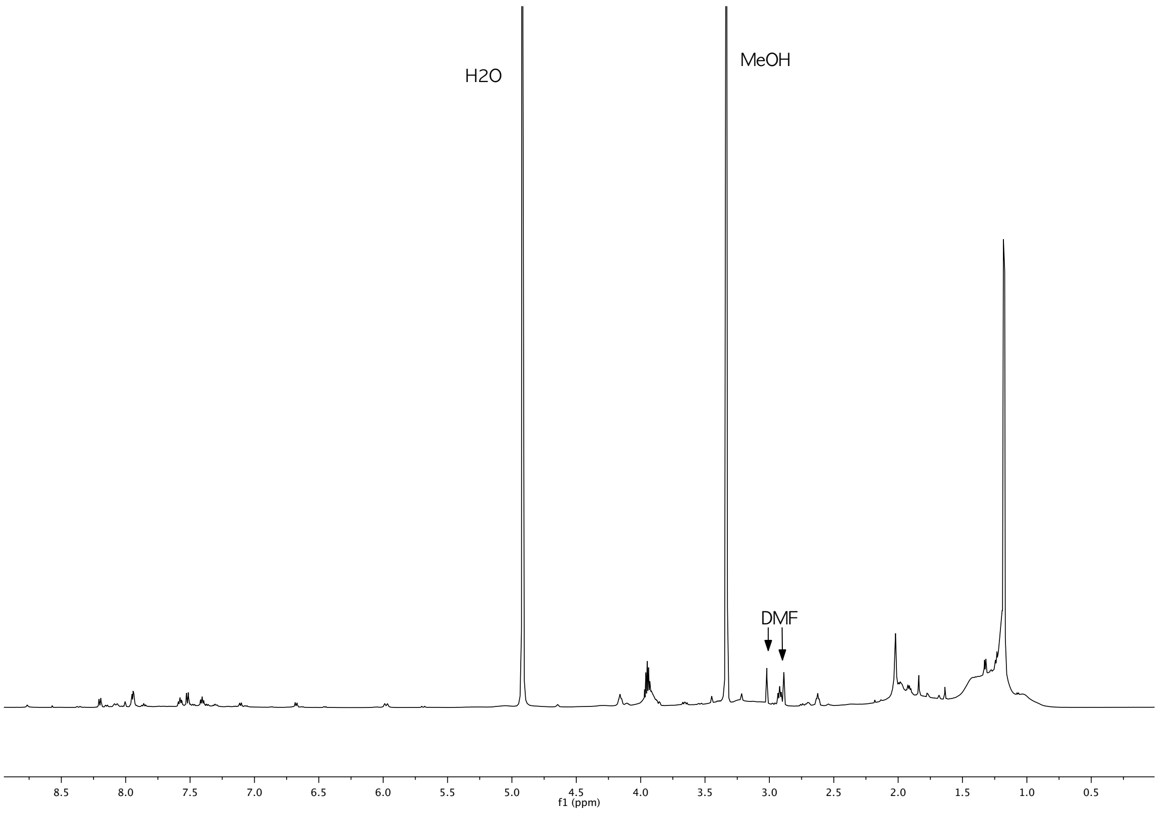

Figure S9. ^{1}H NMR Spectrum (500 MHz, CD_3OD) of IR-pHPMA-a

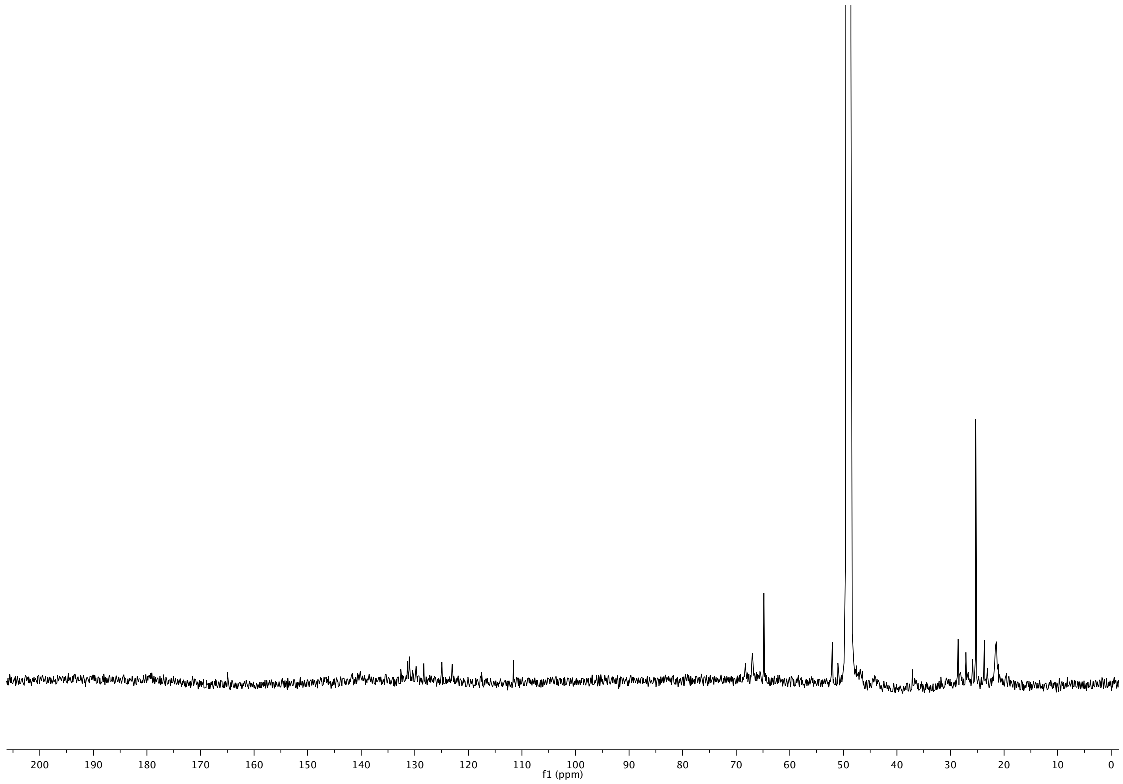

Figure S10. **^{13}C NMR Spectrum (150 MHz, CD_3OD) of IR-pHPMA-a**

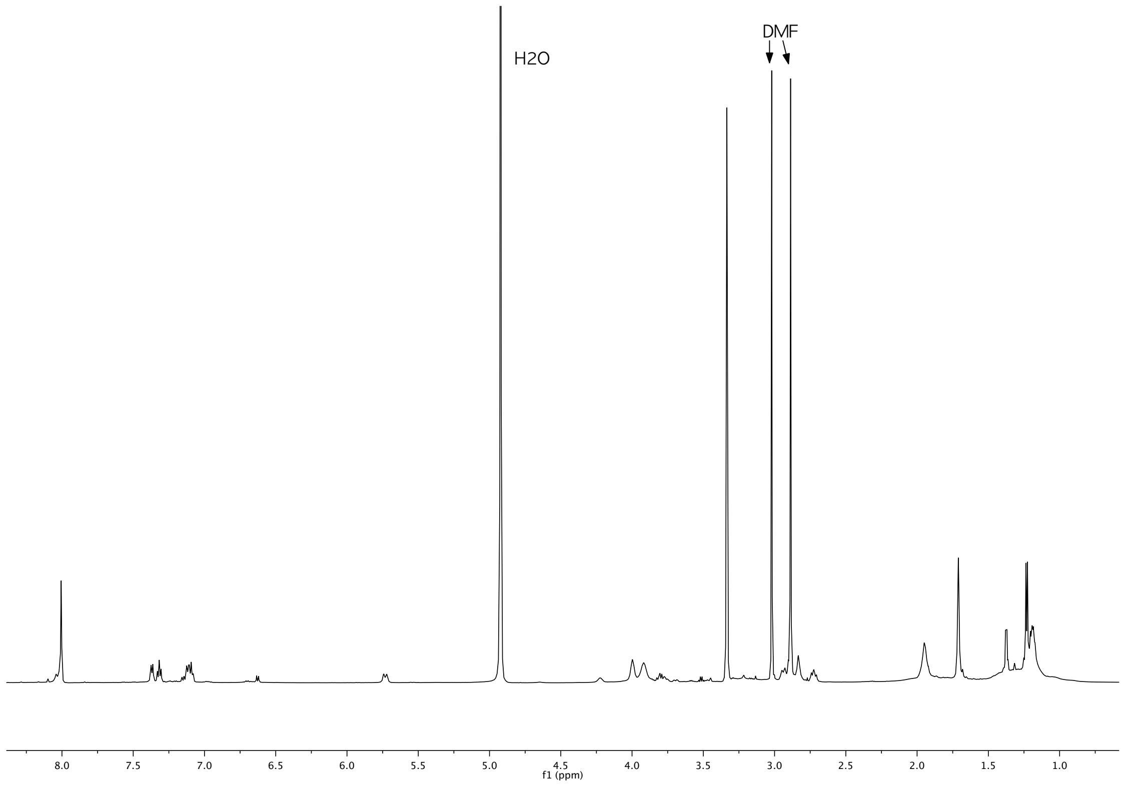

Figure S11. ^{1}H NMR Spectrum (500 MHz, CD_3OD) of IR-pHPMA-b

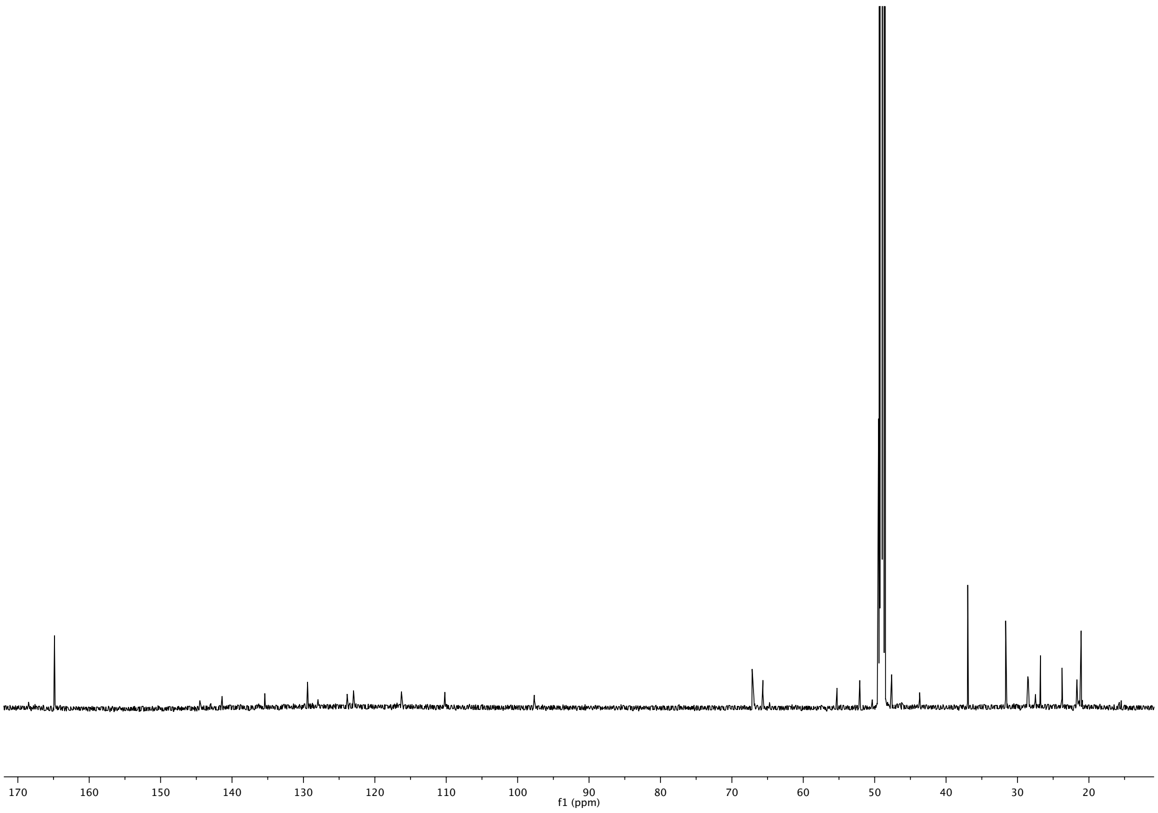

Figure S12. ^{13}**C NMR Spectrum(150 MHz, CD_3OD) of IR-pHPMA-b**

•

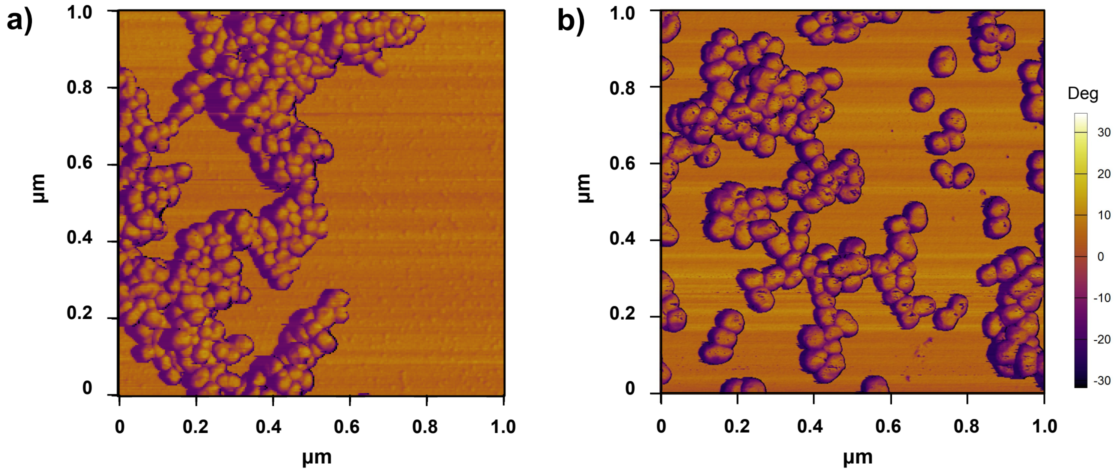

Figure S13. Tapping mode AFM phase images of a) bare gold nanoparticles and b) Au@IR-pHPMA on AP-Mica substrate with an SSS-SEIHR 2 nm high-resolution probe

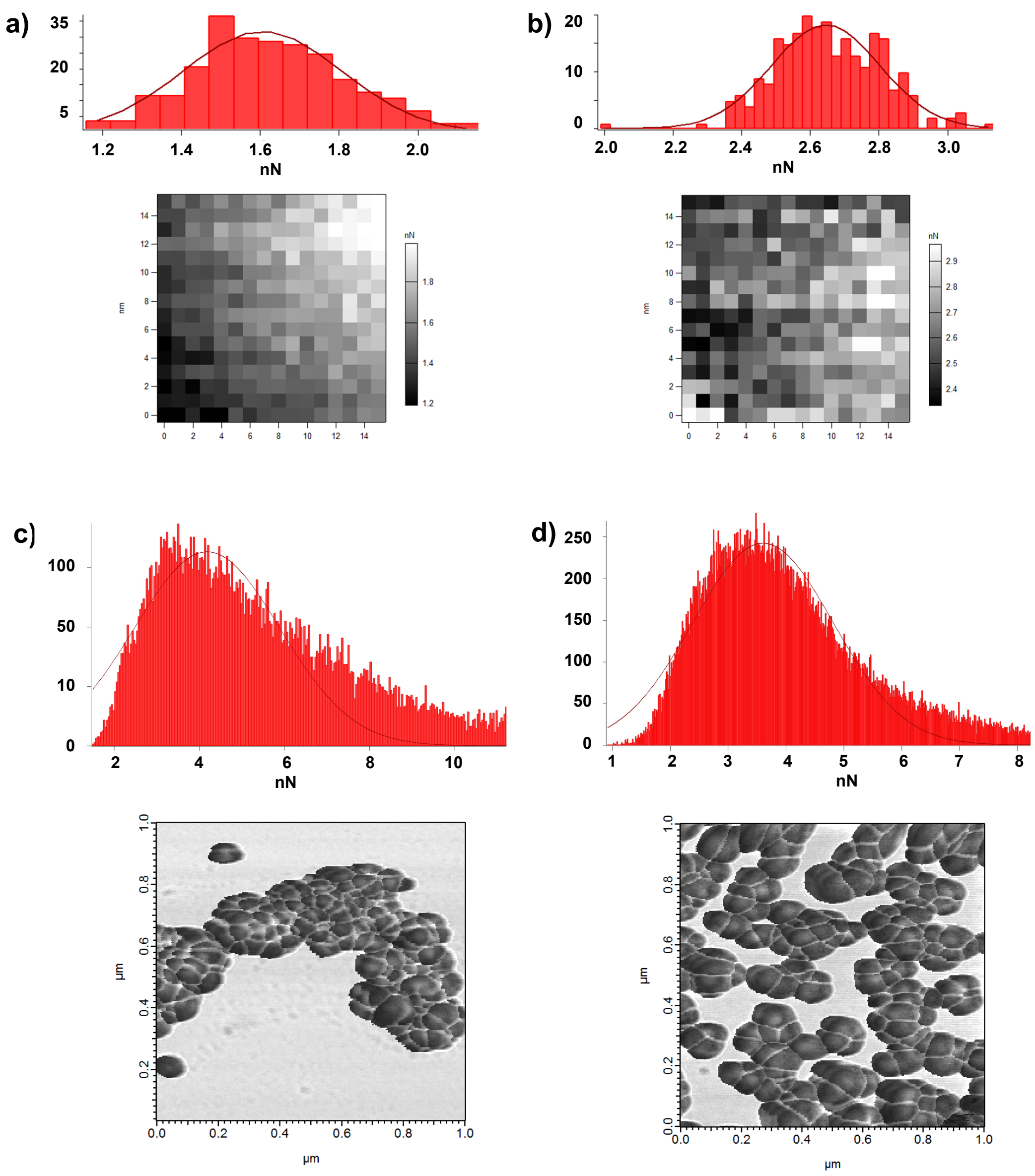

Figure S14. Representative histograms of AFM adhesion measurements and accompanying adhesion maps of a) bare gold nanoparticles and b) Au@IR-pHPMA using a gold AFM probe. Histogram of adhesion force measurements and accompanying adhesion maps for c) bare gold nanoparticles and d) Au@IR-pHPMA using a silicon nitride probe.

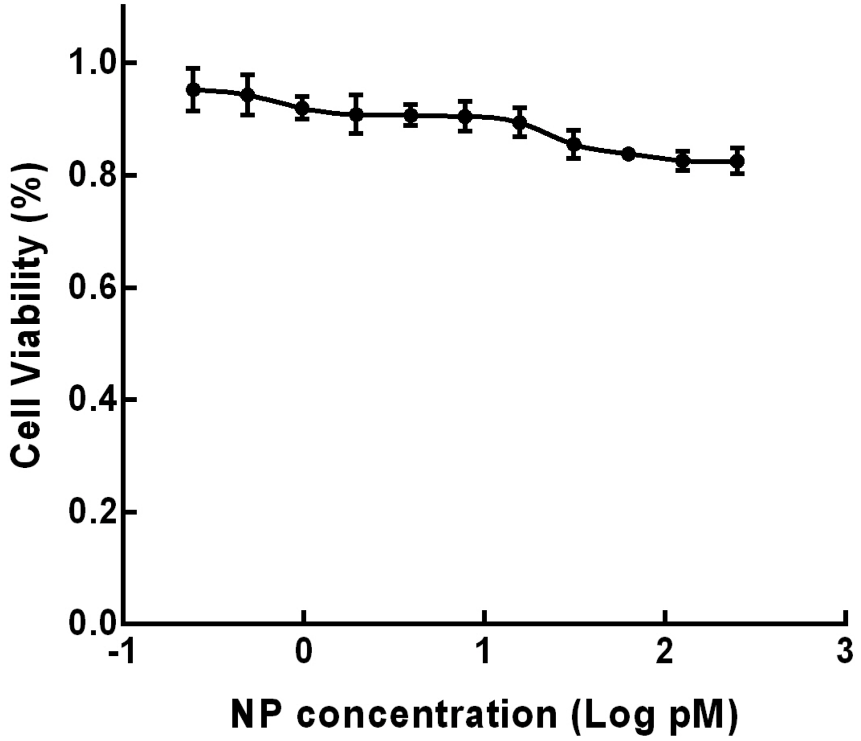

Figure S15. HEK-293 cell viability after 24 h incubation with varying concentrations of Au@IR-pHPMA.

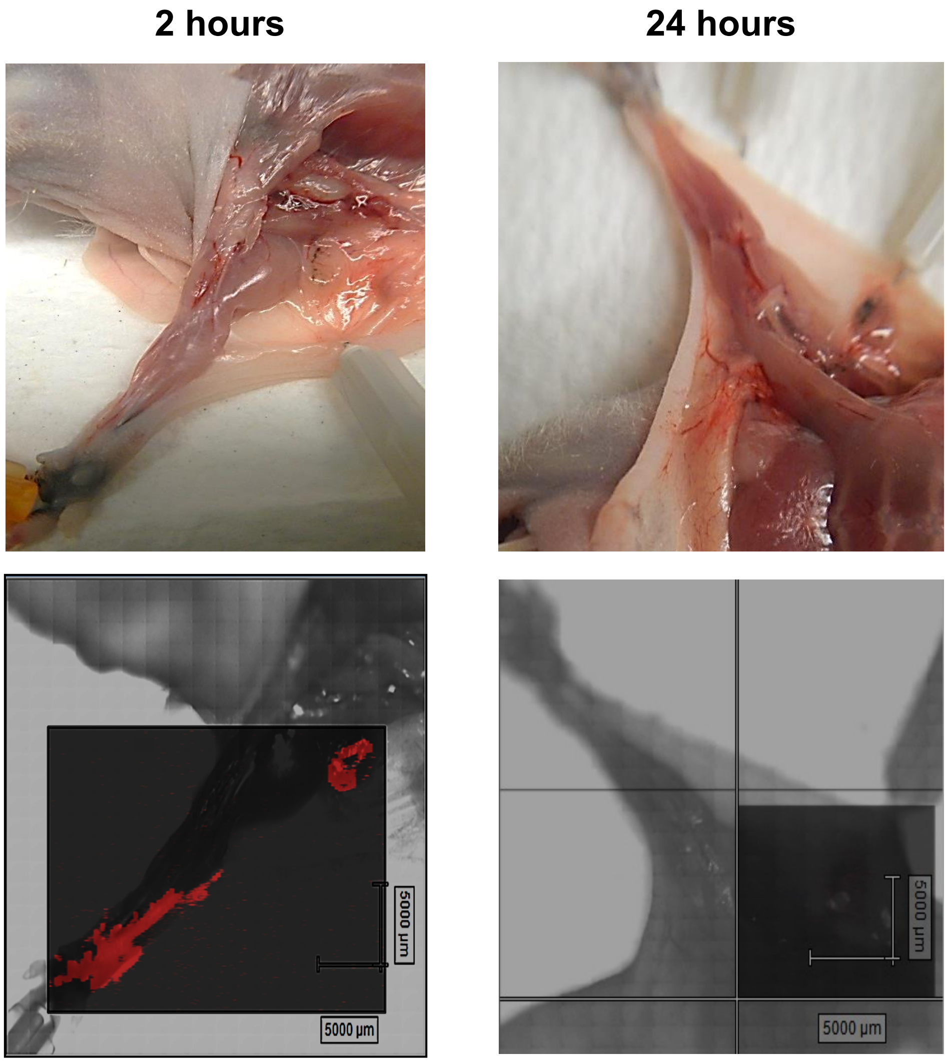

Figure S16. Photographs (top) and SERS images (bottom) of axillary lymph nodes and fore limbs after injection of Au@IR-pHPMA-DTE. As opposed to Au@IR-pHPMA (Figure 2), drastically decreased signal is observed after 24 h.

C. Supplementary References

(1) a) Turkevich, J.; Stevenson, P. C.; Hillier, J. *Discuss. Faraday Soc.* **1951**, *11*, 55. b) Frens, G. *Nature. Phys. Sci.* **1973**, *241*, 20.

(2) a) Jogikalmath, G.; Stuart, J. K.; Pungor, A.; Hlady, V. *Colloids and Surfaces A: Physicochemical and Engineering Aspects* **1999**, *154*, 53. b) Raghavan, D.; Gu, X.; Nguyen, T.; VanLandingham, M.; Karim, A. *Macromolecules* **2000**, *33*, 2573.

(3) Kircher, M. F.; de la Zerda, A.; Jokerst, J. V.; Zavaleta, C. L.; Kempen, P. J.; Mittra, E.; Pitter, K.; Huang, R.; Campos, C.; Habte, F.; Sinclair, R.; Brennan, C. W.; Mellinghoff, I. K.; Holland, E. C.; Gambhir, S. S. *Nat. Med.* **2012**, *18*, 829.

(4) Strekowski, L.; Lipowska, M.; Patonay, G. *J. Org. Chem.* **1992**, *57*, 4578.

(5) Eberhardt, M.; Théato, P. *Macromol. Rapid Commun.* **2005**, *26*, 1488.
